# A successful case of laparoscopic colorectal cancer resection in an elderly patient with factor XI deficiency

**DOI:** 10.1186/s40792-019-0643-4

**Published:** 2019-05-22

**Authors:** Hiroka Kondo, Yasumitsu Hirano, Toshimasa Ishii, Kiyoka Hara, Shintaro Ishikawa, Takuhisa Okada, Nao Obara, Liming Wang, Shigeki Yamaguchi

**Affiliations:** Department of Gastroenterological Surgery, Saitama Medical University International Medical Center, 1397-1 Yamane, Hidaka-shi, Saitama, 350-1298 Japan

**Keywords:** Factor XI deficiency, Fresh frozen plasma, Colon cancer

## Abstract

**Background:**

Congenital XI factor deficiency is a rare disease caused by autosomal recessive inheritance. Clinically, there are few spontaneous hemorrhages, which can cause abnormal bleeding after trauma, surgery, and tooth extraction. We experienced a colon cancer patient with congenital XI factor deficiency who was successfully treated by laparoscopic approach with the administration of the preoperative fresh frozen plasma (FFP).

**Case presentation:**

The patient was an 82-year-old woman who complained of right lower abdominal pain for a period of 2 months with no previous history of abnormal hemostasis. She received colonoscopy and was diagnosed with ascending colon cancer. Preoperative blood tests resulted in prolongation of activated partial thromboplastin time (APTT). After further investigation, factor XI (FXI) activity was found to be abnormal at 3.0% and congenital FXI deficiency was diagnosed. By replenishing FXI by FFP, APTT was improved to 37 s so perioperative abnormal bleeding could be avoided and an operation for ascending colon cancer performed. The patient received laparoscopic ileocolic resection and was discharged on a postoperative day 7 uneventfully.

**Conclusion:**

It is important to detect coagulation disorders such as FXI deficiency during routine preoperative checkups, and it is also important to consider unrecognized coagulation disorders if we encounter unexplained abnormal bleeding after surgery or trauma. In patients who have already been diagnosed with FXI deficiency, appropriate treatment including administration of FFP should be considered before surgery, and laparoscopic approach has a possibility to bring safety outcomes as an effect of the reduction of the intraoperative bleeding.

## Introduction

Factor XI (FXI) deficiency is a very rare autosomal recessive coagulation disorder. FXI deficiency causes a few cases of spontaneous bleeding; however, patients with FXI deficiency are at high risk of bleeding in the trauma and surgery settings. When we treat these patients with invasive procedures, we should pay utmost attention to prevent massive bleeding during or after surgery. Generally, preoperative treatment for FXI deficiency is the use of FXI concentrate. However, FXI preparations are not available in Japan. We present a colon cancer patient with FXI deficiency successfully treated with laparoscopic colectomy and the administration of fresh frozen plasma (FFP).

## Case report

**Fig. 1:**
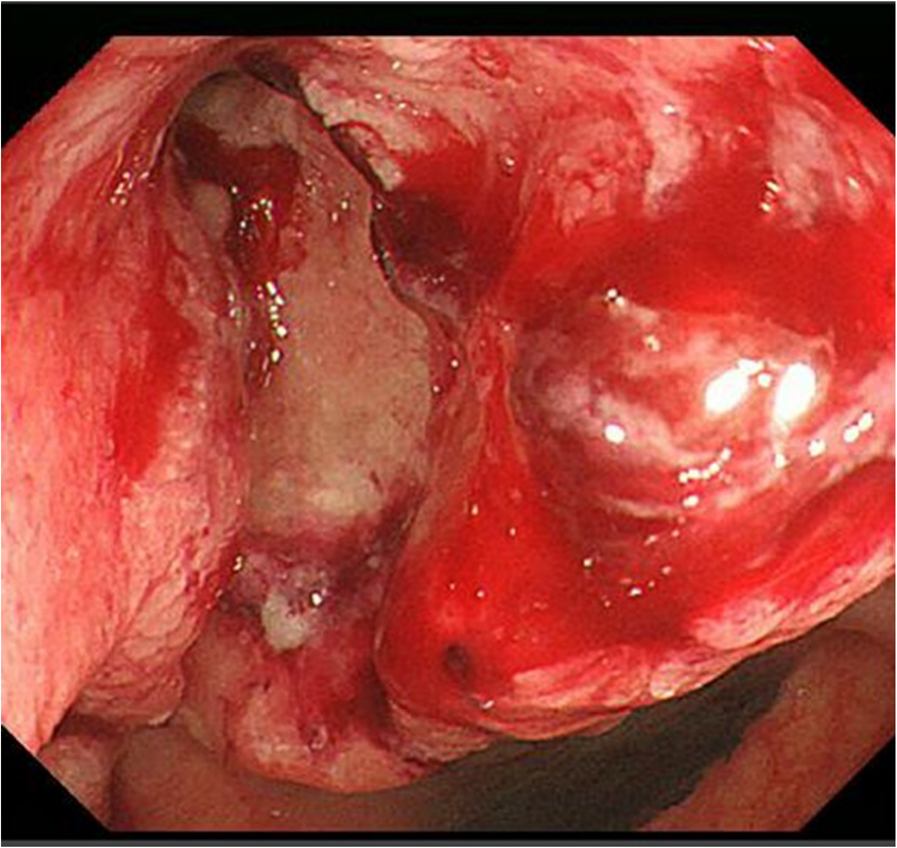
Colonoscopy showed an ascending colon tumor and the colon had become severely stenotic

**Fig. 2:**
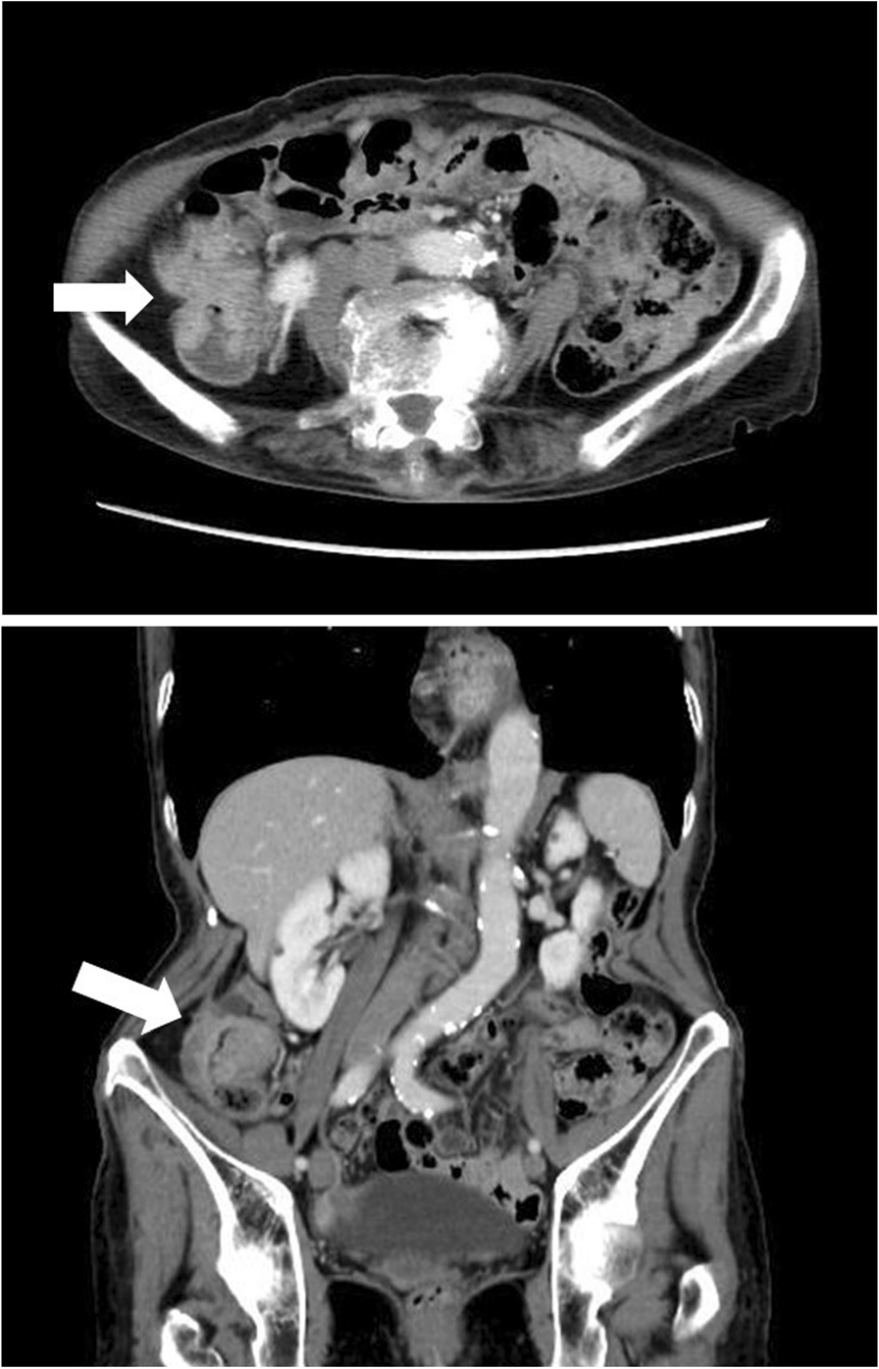
CT: the wall thickness of the ascending colon was observed

**Fig. 3:**
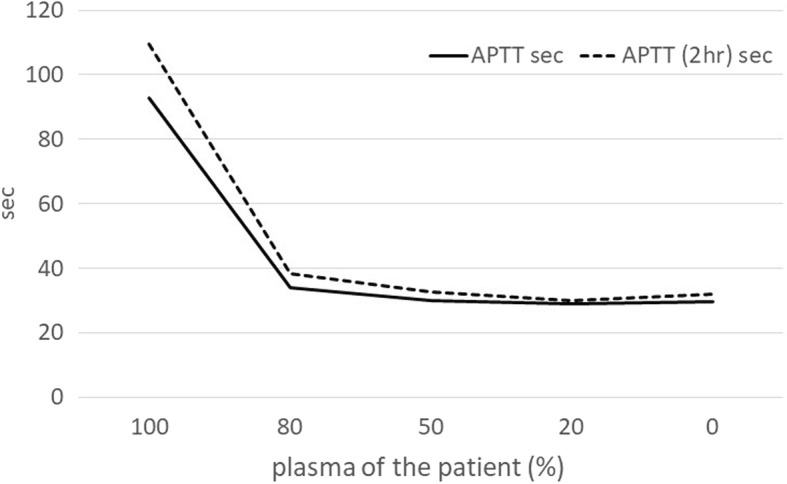
A cross mixing test showed a deficiency pattern

**Fig. 4:**
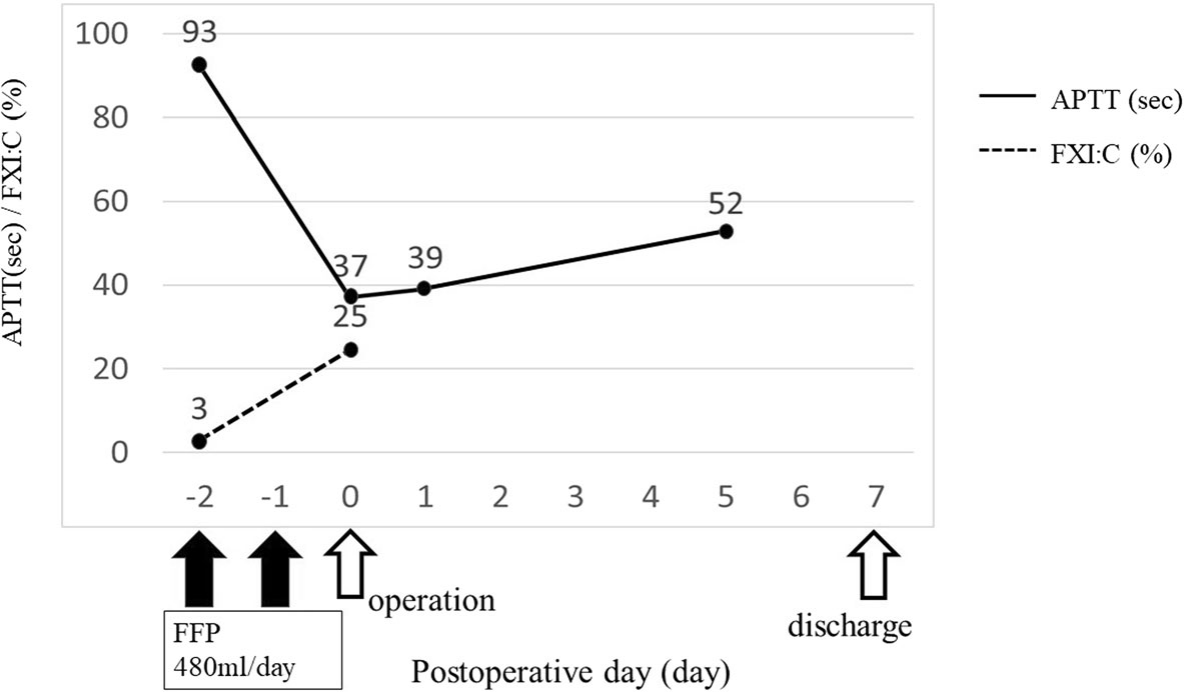
Alterations in APTT and FXI: after transfusion, the APTT improved to 37 s, and FXI also increased from 3% to 25%

An 82-year-old Japanese woman presented at another hospital with right lower abdominal pain of 2 months’ duration. A colonoscopy revealed ascending colon cancer, and she was referred to our hospital. She had no previous history of abnormal bleeding. Her brother had pointed out any abnormalities in his blood coagulation test; however, we could not find the details because he had already died. Her family history except the brother was negative for bleeding tendencies, purpura, and hemophilia. She did not smoke or drink alcohol. She had no allergies. A total colonoscopy showed an ascending colon tumor with severe stenosis (Fig. 1). Computed tomography revealed the wall thickness of the ascending colon (Fig. 2). There were no distant metastases on the liver or lungs. Prolonged activated partial thromboplastin time (APTT 93.9 s) was found incidentally during a routine preoperative examination. However, the prothrombin time (11.2 s), platelet count (480 × 10^3^/L), and liver function test results were all within normal limits. We conducted a cross mixing test, and she was diagnosed with a deficiency pattern (Fig. 3). The activities of factors VIII, IX, XII, and von Willebrand factor were normal. The plasma activity of FXI was 3%, and the diagnosis of FXI deficiency was confirmed. It took about 2 weeks from confirmation of an abnormal APTT time to diagnosis of FXI deficiency. A total of eight units of FFP were transfused for 2 days before the operation. After the transfusion, APTT improved to 37 s, and FXI also increased from 3% to 25% as a result (Fig. 4).

It takes about 3 days to obtain the result of FXI plasma activity in our hospital. We could not know the FXI plasma activity during operation; therefore, we used the APTT level instead of this and decided to do the operation. We performed laparoscopic ileocolic resection with our standard procedure. The operation time was 133 min and the intraoperative blood loss was 10 mL. We did not find the bleeding tendency in intraoperative findings, and we decided not to administrate FFP intraoperatively. The level of APTT on the day after surgery was 39 s. No complications including postoperative bleeding occurred in her postoperative course without additional transfusion of FFP. The final stage was T3N1M0 stage IIIa, according to the Japanese Colorectal Cancer Treatment Code 8th Edition. She was discharged on the seventh postoperative day.

## Discussion

FXI deficiency is a very rare disorder first described by Rosenthal et al. in 1953 [1]. The group with the most reported cases of this type of disorder has been Ashkenazi Jews, with an estimated frequency of homozygotes of around 0.1–0.3% [2]. Only 22 registered FXI deficiency patients were registered in the 2011 Japanese Blood Products Research Organization report [3]. FXI deficiency rarely causes spontaneous bleeding as in patients with other coagulation factor deficiencies such as hemophilia A or B, but it sometimes causes massive bleeding after trauma or surgery [4]. Ragni et al. reported that no patients experienced spontaneous bleeding, including deep muscle hematoma, hemarthrosis, or bleeding into the gastrointestinal tract or retroperitoneal space in 25 patients with FXI deficiency [5]. However, in patients with FXI deficiency, abnormal bleeding has been reported after trauma or surgery [6, 7].

In this case, the prothrombin time was normal and the APTT was prolonged during a routine preoperative examination. We conducted a cross mixing test, and she was diagnosed with a deficiency pattern. The activities of factors VIII, IX, XII, and von Willebrand factor were normal, but the plasma activity of FXI was decreased, and the diagnosis of FXI deficiency was confirmed.

Preoperative treatment of coagulopathy patients aims to supply coagulation factors and may include the use of a coagulation factor concentrate. FXI preparations, however, are not available in Japan, and the first treatment of choice for a patient with FXI deficiency is the administration of FFP. Horio and Yamamoto summarized surgical cases with complications of FXI factor deficiency in Japan [8]. According to this report, only patients who did not receive the administration of fresh blood or FFP before surgery suffered massive postoperative bleeding, and they emphasized the need for preoperative administration of FFP before surgery. Therefore, we believe that it is important to administrate FFP preoperatively not during surgery in patients with FXI factor deficiency, and there was a possibility that the preoperative administration of FFP reduced the amount of bleeding also in this case.

Regarding the levels of FXI, Collins et al. reported a trend for bleeding complications to be associated with lower levels of FXI below 12% [9], and Seligsohn suggested that a safe level of FXI was 45% for major surgery and 30% for minor surgery [10]. Vander Woude et al. revealed that the FXI activity level could be increased to 30–40% by transfusing 10–40 mL/kg/day of FFP [11]. In the present case, the patient’s body weight was 37 kg, and we administrated four units of FFP per day considering the load on circulation dynamics. Her plasma activity of FXI before the administration of FFP was 3% which had a strong possibility to cause perioperative bleeding complications. As a result of transfusion, her preoperative level of FXI increased to 25%, and she overcame her surgery without any bleeding events. This indicated that the preoperative administration of FFP contributed to preventing the postoperative hemorrhagic complications and the safety procedure could be carried out by the laparoscopic approach in spite of FXI level of less than 30%.

There have been many surgical reports of patients with FXI deficiency, but only three surgical reports for colorectal cancer (including the current one) have been described to date in the English literature. A summary of clinical features, FXI activity values, treatments including the administration of FFP, and outcomes in all cases is shown in Table 1 [12, 13]. Age at diagnosis ranged from 42 to 82 years. All three patients were female. The FXI activity value before surgery ranged from less than 1% to 4.2%, far lower than the level that causes bleeding complications. The types of surgery for the three patients were sigmoidectomy, right hemicolectomy, and laparoscopic ileocecal resection, respectively. In all three cases, the administration of FFP was performed before surgery and there were no postoperative hemorrhagic complications. In previously reported cases, laparotomic surgical resection was selected and additional administration of FFP was performed intraoperatively or postoperatively [12, 13]. In our case, we performed laparoscopic resection and successfully treated without the additional administration of FFP. The laparoscopic approach is known to have a clinical benefit to reduce intraoperative blood loss compared to open surgery [14]. We believe that the decrease of intraoperative blood loss owing to the laparoscopic approach had a possibility to bring the reduction of any additional administrations of FFP.

**Table 1:** Summary of the three reported cases of colorectal cancer surgery with factor XI deficiency

Publication year	Author	Sex	Age	FXI activity value	Surgical procedure	Administration of FFP	Postoperative complications
1998	Uen WC et al.	Female	42	< 1%	Sigmoidectomy	Pre-op 4 units Intra-op 2 units	None
2012	Yamaguchi S et al.	Female	74	4.3%	Right hemicolectomy	Pre-op 10 units Post-op 2 units (10 days after op)	None
2018	Our case	Female	82	3%	Laparoscopic ileocolic resection	Pre-op 8 units	None

## Conclusion

It is important to detect coagulation disorders, including FXI deficiency, during routine preoperative checkups, and it is also important to consider unrecognized bleeding disorders such as FXI deficiency if we encounter any unexpected or unexplained massive bleeding after surgery or trauma. In patients in whom FXI deficiency has already been diagnosed, an appropriate treatment including the administration of FFP should be considered before surgery, and laparoscopic approach has a possibility to bring safety outcome as an effect of the reduction of intraoperative bleeding.

## Abbreviations

APTT: Activated partial thromboplastin time
FFP: Fresh frozen plasma
FXI: Factor XI
